# CRP-Cyclic AMP Regulates the Expression of Type 3 Fimbriae via Cyclic di-GMP in *Klebsiella pneumoniae*

**DOI:** 10.1371/journal.pone.0162884

**Published:** 2016-09-15

**Authors:** Ching-Ting Lin, Tien-Huang Lin, Chien-Chen Wu, Lei Wan, Chun-Fa Huang, Hwei-Ling Peng

**Affiliations:** 1 School of Chinese Medicine, China Medical University, Taichung, Taiwan, Republic of China; 2 Division of Urology, Taichung Tzu Chi Hospital, The Buddhist Tzu Chi Medical Foundation, Taichung, Taiwan, Republic of China; 3 Tzu Chi University School of Post-Baccalaureate Chinese Medicine, Hualien, Taiwan, Republic of China; 4 Department of Biological Science and Technology, National Chiao Tung University, Hsinchu, Taiwan, Republic of China; Shanghai Jiao Tong University, CHINA

## Abstract

*Klebsiella pneumoniae* is the predominant pathogen isolated from liver abscesses of diabetic patients in Asian countries. However, the effects of elevated blood glucose levels on the virulence of this pathogen remain largely unknown. Type 3 fimbriae, encoded by the *mrkABCDF* genes, are important virulence factors in *K*. *pneumoniae* pathogenesis. In this study, the effects of exogenous glucose and the intracellular cyclic AMP (cAMP) signaling pathway on type 3 fimbriae expression regulation were investigated. The production of MrkA, the major subunit of type 3 fimbriae, was increased in glucose-rich medium, whereas cAMP supplementation reversed the effect. MrkA production was markedly increased by *cyaA* or *crp* deletion, but slightly decreased by *cpdA* deletion. In addition, the mRNA levels of *mrkABCDF* genes and the activity of P_*mrkA*_ were increased in Δ*crp* strain, as well as the mRNA levels of *mrkHIJ* genes that encode cyclic di-GMP (c-di-GMP)-related regulatory proteins that influence type 3 fimbriae expression. Moreover, the activities of P_*mrkHI*_ and P_*mrkJ*_ were decreased in Δ*lacZ*Δ*crp* strain. These results indicate that CRP-cAMP down-regulates *mrkABCDF* and *mrkHIJ* at the transcriptional level. Further deletion of *mrkH* or *mrkI* in Δ*crp* strain diminished the production of MrkA, indicating that MrkH and MrkI are required for the CRP regulation of type 3 fimbriae expression. Furthermore, the high activity of P_*mrkHI*_ in the Δ*lacZ*Δ*crp* strain was diminished in Δ*lacZ*Δ*crp*Δ*mrkHI*, but increased in the Δ*lacZ*Δ*crp*Δ*mrkJ* strain. Deletion of *crp* increased the intracellular c-di-GMP concentration and reduced the phosphodiesterase activity. Moreover, we found that the mRNA levels of multiple genes related to c-di-GMP metabolism were altered in Δ*crp* strain. These indicate that CRP regulates type 3 fimbriae expression indirectly via the c-di-GMP signaling pathway. In conclusion, we found evidence of a coordinated regulation of type 3 fimbriae expression by the CRP-cAMP and c-di-GMP signaling pathways in *K*. *pneumoniae*.

## Introduction

Cyclic AMP (cAMP) is a well-known second messenger that has a fundamental role in global gene regulation [1]. Its production has been demonstrated to be related to the exogenous glucose level, where bacteria grown in glucose-rich medium showed inhibited cAMP production, whereas bacteria grown in less-preferred carbon sources produced elevated levels of cAMP [1–3]. To balance the intracellular cAMP concentrations, the adenylate cyclase CyaA and the cAMP phosphodiesterase CpdA work in concert to carry out cAMP biosynthesis and degradation, respectively [1, 3–5]. The cellular target for cAMP signaling is the cAMP receptor protein (CRP), which forms a homodimer with cAMP (CRP-cAMP) that then binds to CRP binding sites (TGTGA-N6-TCACA and TGCGA-N6-TCGCA) in the promoter region of DNA in order to activate and control mRNA transcription [6–9]. In *Escherichia coli*, almost 200 operons expression is under CRP-cAMP regulation, which containing genes coding for carbon metabolism and various virulence factors [10–12]. Recently, we found that the biosynthesis of capsular polysaccharides (CPSs) in *Klebsiella pneumoniae* increases in response to exogenous glucose, a process that is regulated by CRP-cAMP [13]. Nevertheless, in *K*. *pneumoniae* pathogenesis, the targets regulated by CRP-cAMP remain largely unknown.

*K*. *pneumoniae* is a gram-negative facultative anaerobes that causes community-acquired diseases including pneumonia, bacteramia, septicamia, and urinary and respiratory tract infections in patients with underlying diseases [14]. In Asian countries, pyogenic liver abscess in diabetic patients is common caused by *K*. *pneumoniae* [15]. Recently, reports of *Klebsiella* liver abscess have also been found in western countries [16]. Pyogenic liver abscess isolated *K*. *pneumoniae* strains often carry heavy CPS that confer not only a mucoid phenotype to the bacterium but also protect the bacteria from phagocytosis and killing by serum factors. The degree of mucoidy in *K*. *pneumoniae* has also been highly related with the successful infection [17, 18]. Besides CPSs, fimbriae are considered as another crucial virulence factor in *K*. *pneumoniae* pathogenesis [19]. Most *K*. *pneumoniae* strains possess type 1 and type 3 fimbriae. In the heavily encapsulated *K*. *pneumoniae* strains, type 3 fimbriae are mainly expressed, whereas type 1 fimbriae are poorly expressed and phase-variable [20, 21]. Type 3 fimbriae, which are encoded by the *mrkABCDF* operon, play an important role in *K*. *pneumoniae* biofilm formation on biotic and abiotic surfaces [22–24]. Biofilm formation is considered to be a key factor in the development of nosocomial infections and increases the bacterial tolerance to antibiotics, which causes problems in medical treatments [25]. Although the diverse regulatory mechanisms of CRP-cAMP on type 1 fimbriae expression and/or biofilm formation have been demonstrated in several bacteria, including *Escherichia coli* [26], *Vibrio cholerae* [27], and *Serratia marcescens* [28], the effect of CRP-cAMP on type 3 fimbriae expression has not been characterized.

Recently, several reports have demonstrated that c-di-GMP is involved in mediating the expression of the two types of fimbriae in *K*. *pneumoniae* [24, 29–33]. Like cAMP, c-di-GMP is also a bacterial second messenger that modulates biofilm formation and controls the expression of virulence genes [34]. The intracellular concentration of c-di-GMP in bacteria is modulated through the activities of di-guanylate cyclases (DGCs) and phosphodiesterases (PDEs) [35, 36]. In *K*. *pneumoniae*, a gene cluster (*mrkHIJ*) adjacent to the type 3 fimbriae operon is involved in the modulation and sensing of c-di-GMP as well as regulation of type 3 fimbriae expression [24, 30–33]. MrkH is a PilZ-domain protein that is able to bind to c-di-GMP to activate type 3 fimbriae expression [32, 33]. In addition, MrkH has autoregulatory activity in response to a high intercellular concentration of c-di-GMP to further activate type 3 fimbriae expression [31]. MrkI has been shown to be a LuxR-type transcriptional regulator, activating its own operon and regulating type 3 fimbriae expression [24]. MrkJ possesses an EAL domain that functions as a PDE to hydrolyze c-di-GMP as well as to repress type 3 fimbriae expression [30]. Reverse-transcription PCR analysis revealed that *mrkHIJ* could be transcribed as a polycistronic mRNA, but *mrkJ* is activated independently [24]. Although the important roles of MrkH, MrkI, and MrkJ in regulating type 3 fimbriae expression is well established, the regulation of *mrkHIJ* is still unknown.

The coordination of various nucleotide second messenger signaling pathway to control physiological function and virulence factor expression in response to environmental stimulus is common [37–39]. In *E*. *coli*, c-di-GMP, cAMP, and (p)ppGpp signaling pathways are coordinated to regulate the bacterial mobility and expression of curli [40, 41]. In *V*. *cholera*, the regulation of the biofilm formation, toxin production, and fimbriae expression is connected by c-di-GMP and cAMP signaling pathways [27, 42, 43]. In *Pseudomonas aeruginosa*, the expression of acute virulence genes is regulated by the cross-talk between c-di-GMP and cAMP signaling pathways [44]. However, whether cAMP and c-di-GMP signaling pathways also coordinated to regulate the virulence gene expression in *K*. *pneumoniae* remains unknown.

Herein, we found that exogenous glucose could activate type 3 fimbriae expression via modulation of the cAMP level in *K*. *pneumoniae* CG43. CRP regulation of type 3 fimbriae expression is required for MrkH and MrkI activation. In addition, CRP affects the expression of c-di-GMP-related genes to influence the intracellular concentration of c-di-GMP and PDE activity. Taken together, we provide evidence of the coordination of the CRP-cAMP and c-di GMP-signaling pathways in modulating type 3 fimbriae expression in *K*. *pneumoniae* CG43.

## Materials and Methods

### Bacterial strains, plasmids, and media

Bacterial strains and plasmids used in this study are listed in S1 Table. Primers used in this study are list in S2 Table. Bacterial were routinely cultured at 37°C in Luria-Bertani (LB) medium supplemented with appropriate antibiotics. The antibiotics used include ampicillin (100 μg/ml), kanamycin (25 μg/ml), streptomycin (500 μg/ml), and tetracycline (12.5 μg/ml). Glucose and cAMP were dissolved by distilled water and filter sterilized. To prepare LB medium with various stimuli, autoclaved 2X LB medium was diluted by sterile water containing different concentrations of glucose and/or cAMP of the same volume.

### Construction of the gene-deletion mutants

Specific gene deletion was introduced into *K*. *pneumoniae* CG43S3 or CG43S3-derived strains using an allelic exchange strategy as previously described [45]. The pKAS46 system was used in the selection of the mutants [46], and the mutations were respectively confirmed by PCR and Southern hybridization (data not shown).

### Quantitative reverse-transcription PCR (qRT-PCR)

Total RNA extraction, reverse transcription of isolated mRNA to cDNA, qRT-PCR, and data analysis were performed as described in detail previously [47]. Primers and probes were designed for selected target sequences using Universal ProbeLibrary Assay Design Center (Roche-applied science) and shown in S2 Table. Relative gene expressions were quantified using the comparative threshold cycle 2^-ΔΔCT^ method with 23S rRNA as the endogenous reference.

### Measurement of promoter activity

The promoter region of *mrkHI* and *mrkJ* was PCR-amplified with primer pair GT288/GT289 and GT284/GT285, respectively, and the amplicons were then cloned into placZ15 to generate pmrkHIZ15 and pmrkJZ15. The promoter-reporter plasmids, pmrkAZ15, pmrkHZ15, and pmrkJZ15, were mobilized into *K*. *pneumoniae* strains by electroporation, respectively. The β-galactosidase activity of logarithmic phase bacteria was measured as previously described [48].

### Western blotting

*K*. *pneumoniae* cultures were collected by centrifugation, re-suspended in PBS, and lysed by sonication. The bacterial total proteins were quantified by Bradford protein assay (Biorad), separated by SDS-PAGE (approximately 5 μg per lane), and transferred to PVDF membrane. After incubation with the corresponding antisera, membranes were visualized with an enhanced chemiluminescence ECL western blotting luminal reagent (PerkinElmer, Wellesley, MA, USA), and the signal was collected by ImageQuant LAS 4000 mini (GE Health, USA).

### Intracellular concentration of c-di-GMP

As previous study, the intracellular concentration of c-di-GMP was extracted in *K*. *pneumoniae* using heat and ethanol precipitation [49]. The lyophilized samples were resuspended in distill water and further were measured the c-di-GMP level by a ELISA kit (Wuhan EIAab Science Co., Ltd). The c-di-GMP concentration was normalized by total protein concentration. The relative c-di-GMP content of Δ*crp* strain against WT strain was shown.

### PDE activity

The PDE activity of the crude extracts in *K*. *pneumoniae* CG43S3 WT and mutant strains was performed as previous study by using *bis*-*p*-nitrophenyl phosphate (bis-*p*NPP) [30]. Briefly, 10 μg of total protein in assay buffer (50 mM Tris-HCl, 1 mM MnCl 2 [pH 8.5]) supplemented with 5 mM bis-*p*NPP at 37°C for 5 min. The PDE activity was determined by measuring the release of *p*-nitrophenol at 410 nm. Percentage of PDE activity was quantified by dividing the OD410 of WT strain.

### Statistical method

The results of qRT-PCR analysis, and promoter activity, the intracellular concentration of c-di-GMP, and PDE activity measurement were performed in triplicate. The results are presented as the mean and standard deviation. Differences between groups were evaluated by an unpaired *t*-test. Values of *P*<0.05 and *P*<0.01 were considered statistically significant difference.

## Results

### Glucose and cAMP affect type 3 fimbriae expression

**Fig 1:**
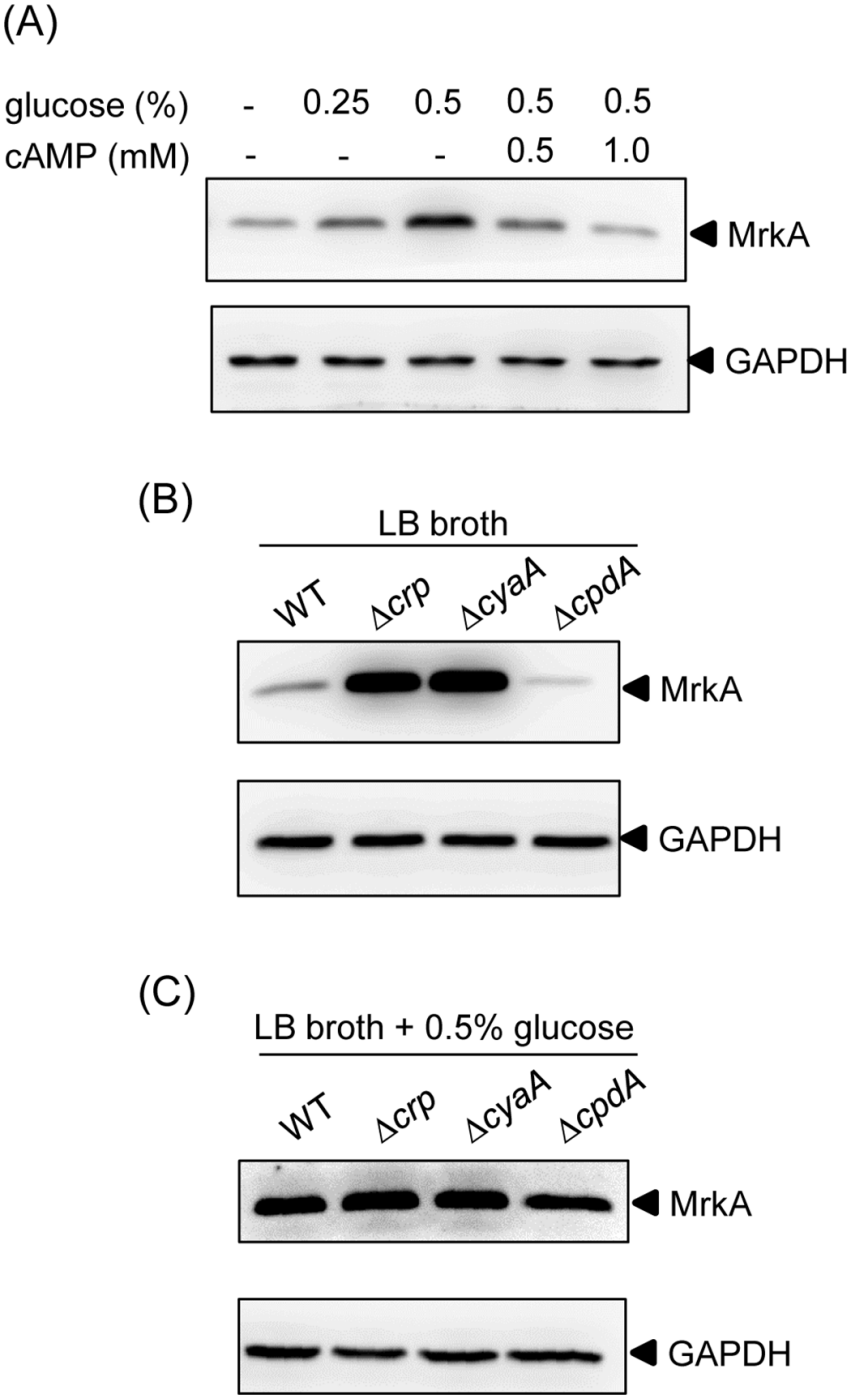
Glucose and cAMP affects the type 3 fimbriae expression of *K*. *pneumoniae* CG43S3. (A) *K*. *pneumoniae* CG43S3 was grown overnight at 37°C with agitation in LB broth supplemented with glucose and cAMP as indicated. *K*. *pneumoniae* CG43S3 WT, Δ*crp*, Δ*cyaA*, and Δ*cpdA* strains was grown overnight at 37°C with agitation in LB broth alone (B) or LB broth with 0.5% glucose (C) at 37°C with agitation to observe the type 3 fimbriae expression by Western blot analysis against MrkA (the upper panel) and GAPDH antiserum (the lower panel, for internal control). The MrkA and GAPDH proteins are indicated by an arrow, respectively.

To analyze whether exogenous glucose and cAMP affect *K*. *pneumoniae* type 3 fimbriae expression, the bacterium was grown in LB broth supplemented with increasing amounts of glucose, and the level of MrkA (the major subunit of type 3 fimbriae) expression was quantified. As shown in Fig 1A, MrkA production increased with increasing amounts of glucose in the LB broth. Based on the intracellular cAMP concentration was reduced by the presence of glucose in the growth medium [1, 2], increasing amounts of exogenous cAMP were added to LB broth supplemented with 0.5% glucose to observe the production of MrkA. The addition of exogenous cAMP repressed the effect of glucose on MrkA production, suggesting that exogenous glucose can activate *K*. *pneumoniae* type 3 fimbriae expression through a reduction of the cAMP level. Besides, the addition of 0.5, 1, or 2 mM cAMP to LB broth did not apparently affect the production of MrkA (data not shown). To further investigate the role of the CRP-cAMP signaling pathway in type 3 fimbriae expression, gene-deletion mutants Δ*crp*, Δ*cyaA*, and Δ*cpdA* were generated and analyzed for the effect of the respective missing genes on MrkA production. As shown in Fig 1B, compared with the wild-type (WT) strain, we found that MrkA production increased in Δ*crp* and Δ*cyaA* strains, but slightly decreased in Δ*cpdA* strain. However, when the bacteria were grown in LB broth supplemented with 0.5% glucose, the deleting effect of *crp* or *cyaA* on the MrkA production was diminished (Fig 1C), which may due to the inactivated state of CRP-cAMP signaling pathway in glucose-rich conditions. Deletion of *cpdA* also caused a slight reduction of MrkA production (Fig 1C). These results suggested that the CRP-cAMP signaling pathway is downstream of glucose treatment to regulate the expression of type 3 fimbriae in *K*. *pneumoniae*.

### CRP acts as a transcriptional repressor of type 3 fimbriae gene expression

**Fig 2:**
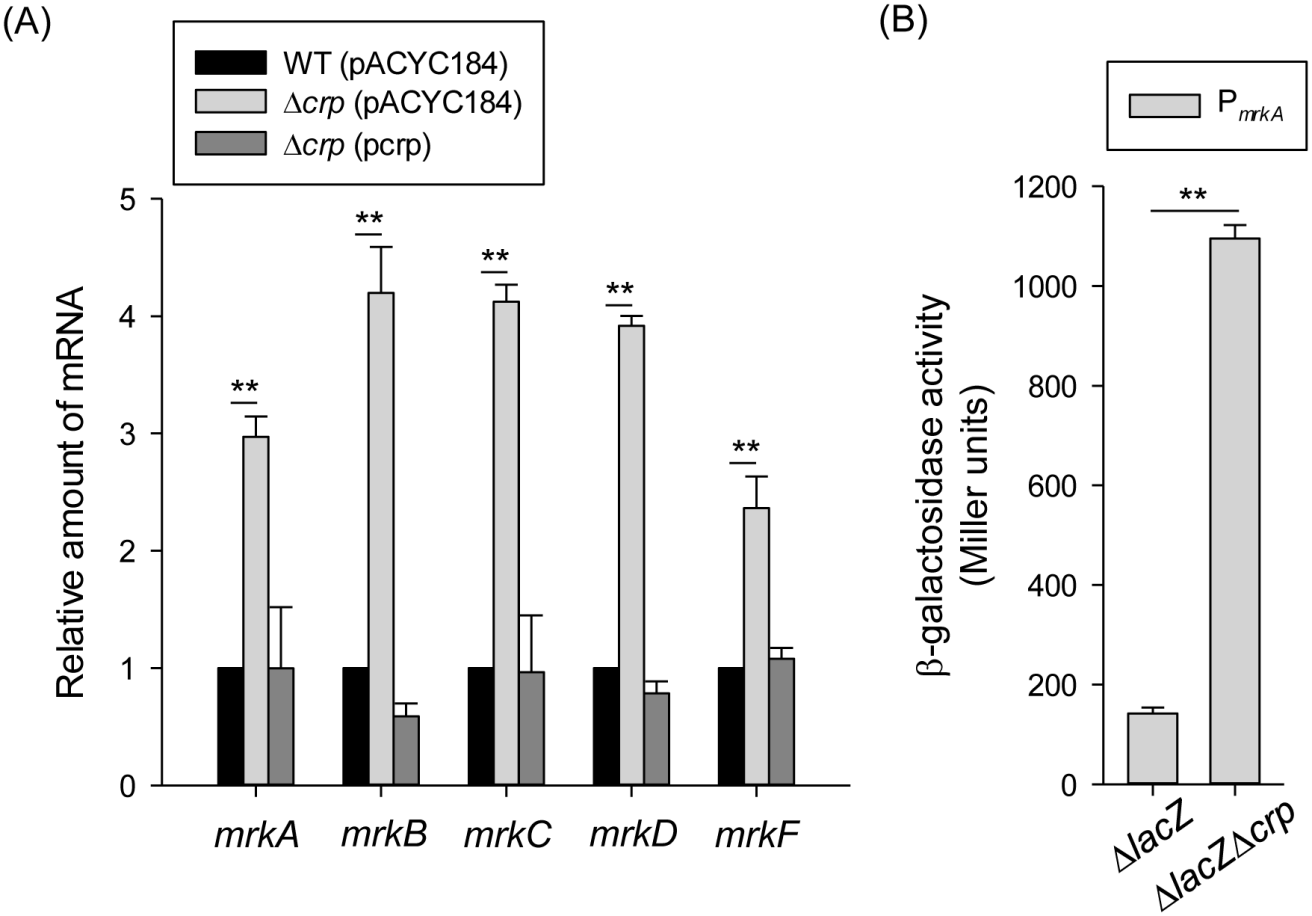
CRP-cAMP affects the type 3 fimbriae expression. (A) qRT-PCR analyses of the *mrkA*, *mrkB*, *mrkC*, *mrkD*, and *mrkF* expressions for WT (pACYC184), Δ*crp* (pACYC184), and Δ*crp* (pcrp) strains in LB medium. (B) β-galactosidase activities of *K*. *pneumoniae* CG43S3Δ*lacZ* and the isogenic strain (Δ*lacZ*Δ*crp*) carrying the reporter plasmid pmrkAZ15 (P_*mrkA*_::*lacZ)* were determined using log-phase cultures grown in LB medium. The results are representative of three independent experiments. Error bars indicate standard deviations. *P < 0.05 and ** *P* < 0.01 compared to WT (pACYC184) (A) and CG43S3Δ*lacZ* (B) strain.

To study the regulatory role of CRP in type 3 fimbriae expression, the effect of *crp* deletion on the mRNA levels of the type 3 fimbriae gene cluster *mrkABCDF* was measured by qRT-PCR. As shown in Fig 2A, in the Δ*crp* strain carrying the empty vector control (pACYC184), the mRNA levels of *mrkABCDF* were increased relative to those of the WT strain. Introduction of the plasmid complement p*crp* into Δ*crp* strain reversed the effect of the deletion. To further investigate whether CRP acts as a transcriptional repressor of *mrkA*, a plasmid carrying P_*mrkA*_ fused with the *lacZ* reporter gene was constructed and introduced into the Δ*lacZ* and Δ*lacZ*Δ*crp* strains, respectively. As shown in Fig 2B, the P_*mrkA*_ activity was significantly higher in Δ*lacZ*Δ*crp* stain than in Δ*lacZ* strain, suggesting that CRP represses type 3 fimbriae expression at the transcriptional level.

### MrkH, MrkI, and MrkJ are involved in the CRP regulation

**Fig 3:**
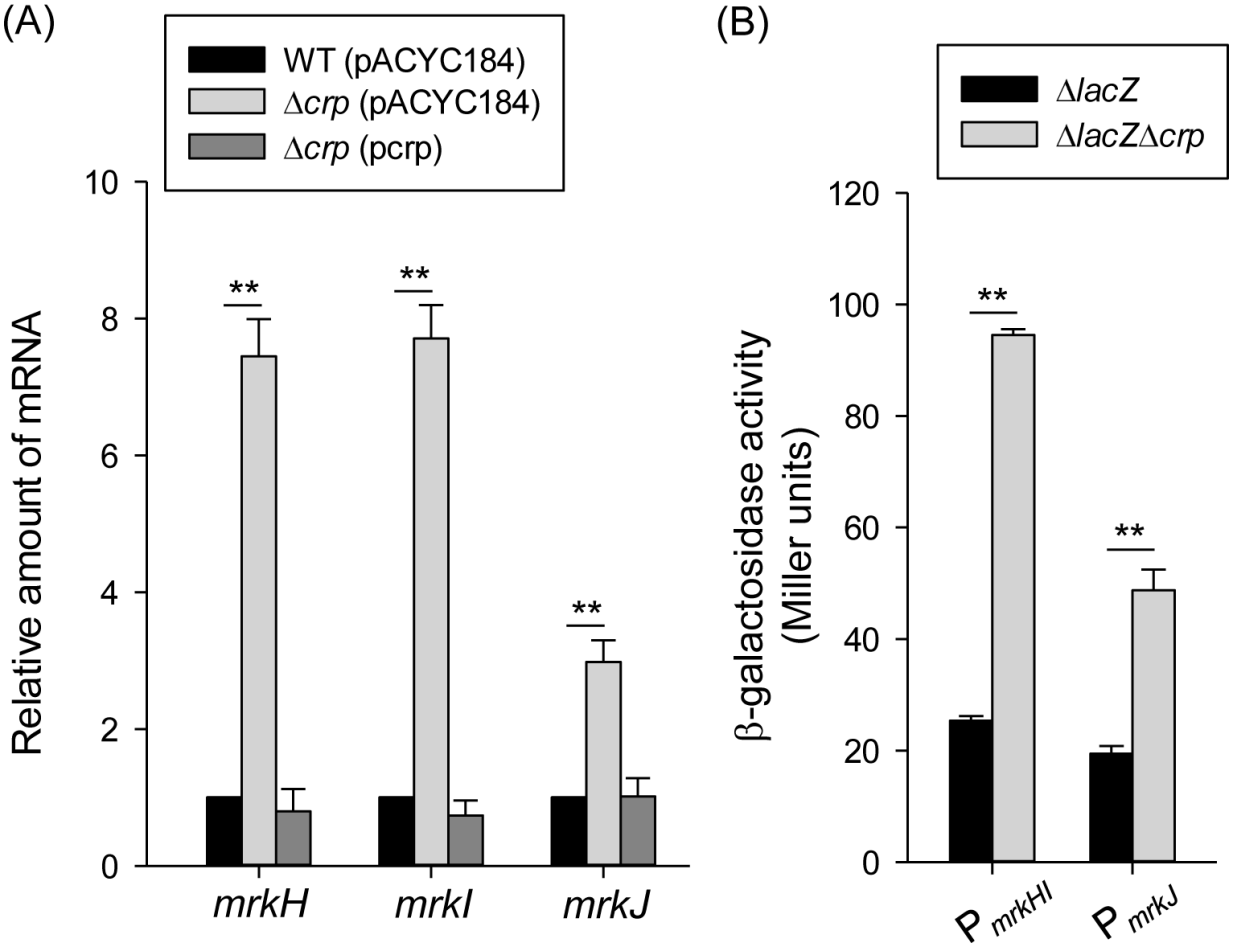
CRP represses the *mrkHIJ* expression. (A) qRT-PCR analysis of *mrkH*, *mrkI*, and *mrkJ* expression was measured in WT (pACYC184), Δ*crp* (pACYC184), and Δ*crp* (pcrp) strains in LB medium. (B) β-galactosidase activities of *K*. *pneumoniae* CG43S3Δ*lacZ* and the isogenic strain (Δ*lacZ*Δ*crp*) carrying the reporter plasmids pmrkHIZ15 (P_*mrkHI*_::*lacZ*) and pmrkJZ15 (P_*mrkJ*_::*lacZ*) were determined using log-phase cultures grown in LB medium. The results are representative of three independent experiments. Error bars indicate standard deviations. *P < 0.05 and ** *P* < 0.01 compared to WT (pACYC184) (A) and CG43S3Δ*lacZ* (B) strain.

To further study the regulatory mechanism of CRP-cAMP in *mrkA* gene transcription, the sequences of the *E*. *coli* CRP binding sites (TGTGA-N6-TCACA and TGCGA-N6-TCGCA) were used to search for the promoter sequence of the *mrkA* gene. However, no typical CRP binding site was found in the promoter sequence of *mrkA*, suggesting that CRP regulates *mrkA* transcription indirectly. According to previous studies, a c-di-GMP-related gene cluster (i.e., *mrkHIJ*) adjacent to the type 3 fimbriae operon has been demonstrated to regulate type 3 fimbriae expression [24, 25]. To further investigate whether *mrkHIJ* are involved in the CRP regulon, the effect of *crp* deletion on the mRNA levels of these genes was determined by qRT-PCR. Compared with the WT[pACYC184] strain, the mRNA levels of *mrkH*, *mrkI*, and *mrkJ* were markedly increased in Δ*crp*[pACYC184] strain (Fig 3A). The introduction of p*crp* into Δ*crp* restored the levels of *mrkH*, *mrkI*, and *mrkJ* to the same levels as observed in the WT[pACYC184] strain. This indicates that CRP represses the expression of *mrkH*, *mrkI*, and *mrkJ*. Furthermore, the activities of the promoters P_*mrkHI*_ and P_*mrkJ*_ were also repressed by CRP (Fig 3B), confirming that CRP acts a transcriptional repressor of *mrkHIJ* expression.

### Regulation of CRP in type 3 fimbriae expression is dependent on MrkH and MrkI

**Fig 4:**
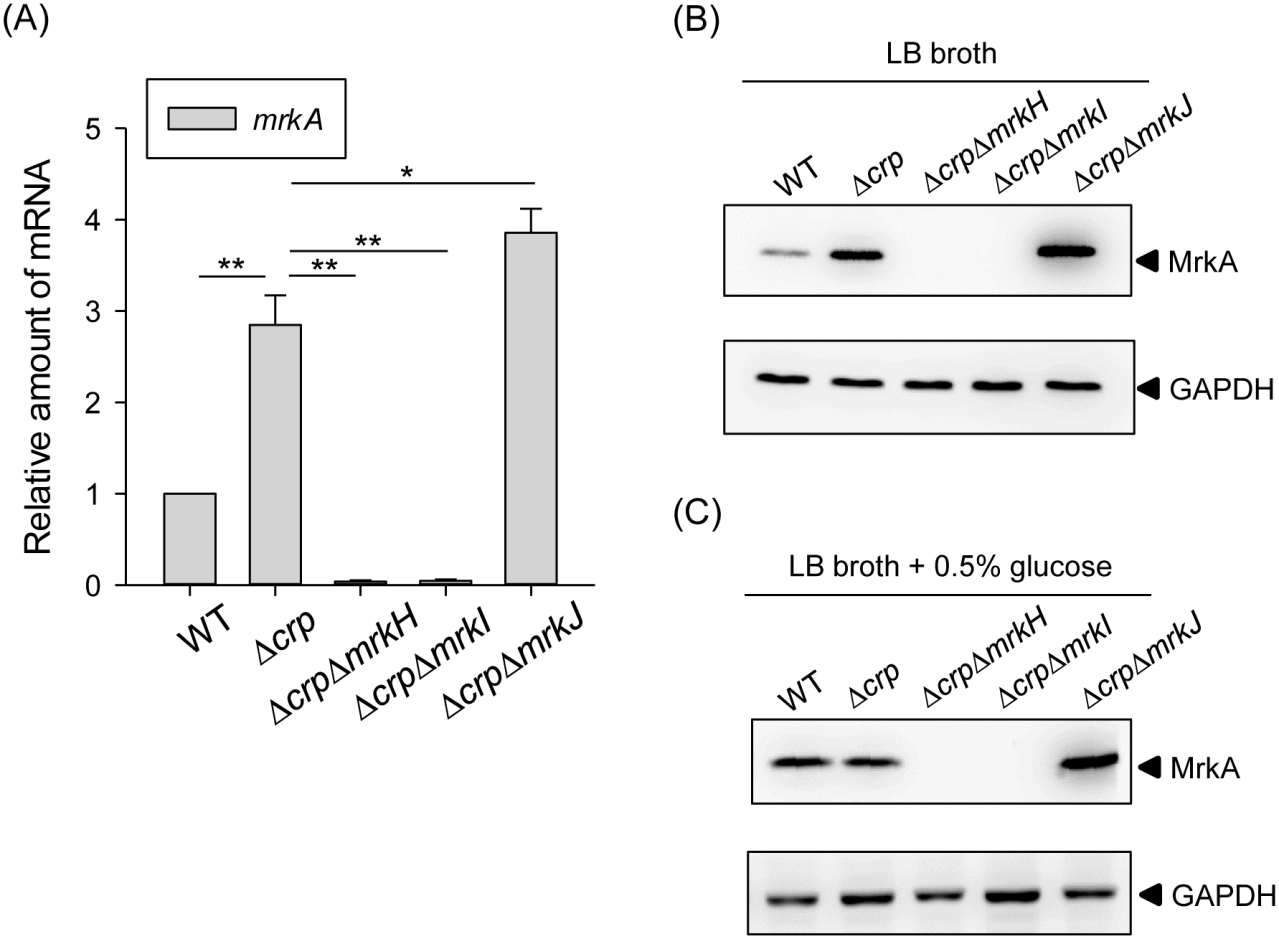
MrkH and MrkI are required for repression of CRP in type 3 fimbriae. (A) qRT-PCR analysis of *mrkA* expression was measured in WT, Δ*crp*, Δ*crp*Δ*mrkH*, Δ*crp*Δ*mrkI*, and Δ*crp*Δ*mrkJ* strains in LB medium. The results are representative of three independent experiments. Error bars indicate standard deviations. *P < 0.05 and ** *P* < 0.01 compared to the indicated group. The MrkA expression of WT, Δ*crp*, Δ*crp*Δ*mrkH*, Δ*crp*Δ*mrkI*, and Δ*crp*Δ*mrkJ* strains was determined in LB broth without (B) or with 0.5% glucose (C) by Western blot analysis against MrkA (the upper panel) and GAPDH antiserum (the lower panel, for internal control). The MrkA and GAPDH proteins are indicated by an arrow, respectively.

Several studies have demonstrated that MrkH and MrkI activate type 3 fimbriae expression in *K*. *pneumoniae*, whereas MrkJ represses type 3 fimbriae expression via the PDE degradation of c-di-GMP [24, 30, 32]. To verify the roles of MrkH, MrkI, and MrkJ in the CRP regulation of type 3 fimbriae expression, the mRNA level of *mrkA* was determined in the WT, Δ*crp*, Δ*crp*Δ*mrkH*, Δ*crp*Δ*mrkI*, and Δ*crp*Δ*mrkJ* strains. As shown in Fig 4A, compared with strain Δ*crp*, the mRNA level of *mrkA* was reduced in Δ*crp*Δ*mrkH* and Δ*crp*Δ*mrkI* strains, but increased in Δ*crp*Δ*mrkJ* strain, indicating that the deletion of *mrkJ* in Δ*crp* strain had a positive effect on *mrkA* expression. Furthermore, the MrkA production levels confirmed the effects of *mrkH*, *mrkI*, and *mrkJ* deletions in the Δ*crp* strain (Fig 4B), indicating that MrkH and MrkI are required for type 3 fimbriae expression and may therefore be repressed by CRP. Besides, similar results could be observed when the bacteria were grown in LB broth supplemented with 0.5% glucose (Fig 4C), confirming that MrkH and MrkI are required for the type 3 fimbriae expression in response to exogenous glucose stimuli. In addition, we suggest that CRP also affects the intracellular concentration of c-di-GMP to control type 3 fimbriae expression.

### CRP represses the auto-regulatory activities of MrkH and MrkI

**Fig 5:**
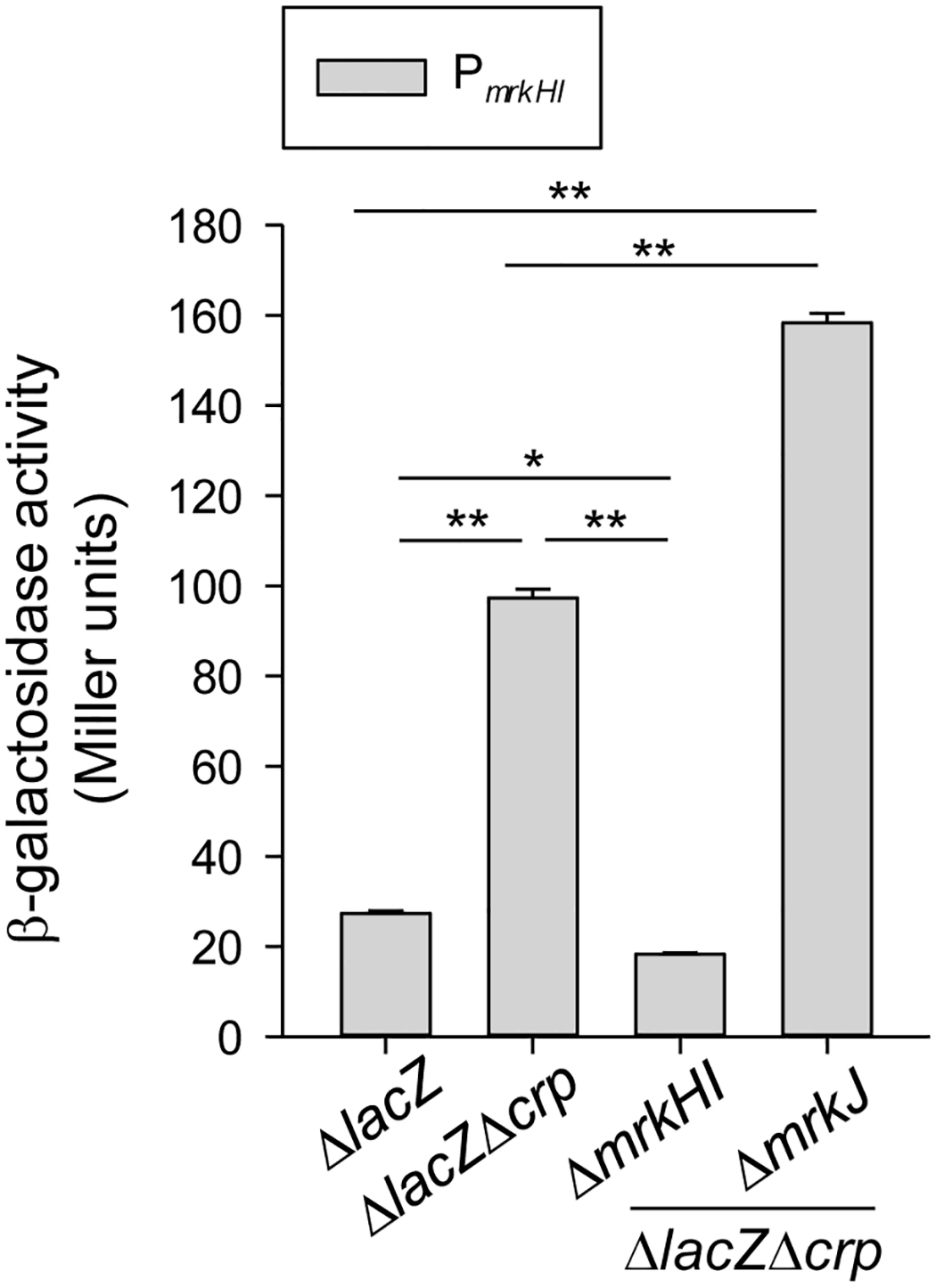
CRP represses the auto-regulatory activities of MrkH and MrkI. β-galactosidase activities of *K*. *pneumoniae* CG43S3Δ*lacZ* and the isogenic strains (Δ*lacZ*Δ*crp*, Δ*lacZ*Δ*crp*Δ*mrkHI*, and Δ*lacZ*Δ*crp*Δ*mrkJ*) carrying the reporter plasmid pmrkHIZ15 (P_*mrkHI*_::*lacZ*) was determined using log-phase cultures grown in LB medium. The results are representative of three independent experiments. Error bars indicate standard deviations. *P < 0.05 and ** *P* < 0.01 compared to the indicated group.

Until now, the regulation of *mrkHI* in *K*. *pneumoniae* has remained largely unknown. As suggested above, the expression of *mrkHI* appears to be repressed by CRP. However, since no typical CRP binding site was found in the sequence upstream of *mrkHI*, the CRP repression of *mrkHI* transcription is likely to be indirect. According to previous studies, MrkH and MrkI could exert autoregulatory activity on their own promoter [24, 31]. Therefore, we postulated that the high activity of promoter P_*mrkHI*_ in Δ*crp* strain is due to autoactivation by MrkH and MrkI. To demonstrate this possibility, the activity of P_*mrkHI*_ was determined in Δ*lacZ*, Δ*lacZ*Δ*crp*, and Δ*lacZ*Δ*crp*Δ*mrkHI* strains. As shown in Fig 5, the absence of *mrkHI* in Δ*lacZ*Δ*crp*Δ*mrkHI* strain resulted in lower P_*mrkHI*_ activity than that in Δ*lacZ*Δ*crp* strain. In addition, to confirm that the expression of *mrkHI* is c-di-GMP dependent, gene *mrkJ* was deleted in Δ*lacZ*Δ*crp* strain and the activity of P_*mrkHI*_ was observed. As expected, P_*mrkHI*_ activity was increased in Δ*lacZ*Δ*crp*Δ*mrkJ* strain relative to Δ*lacZ*Δ*crp* strain, confirming that the expression of *mrkHI* is c-di-GMP-dependent. Taken together, this indicates that CRP represses the autoactivation activity of MrkH and MrkI to further lead to the reduction of type 3 fimbriae expression.

### CRP affects the intracellular c-di-GMP concentration and PDE activity

**Fig 6:**
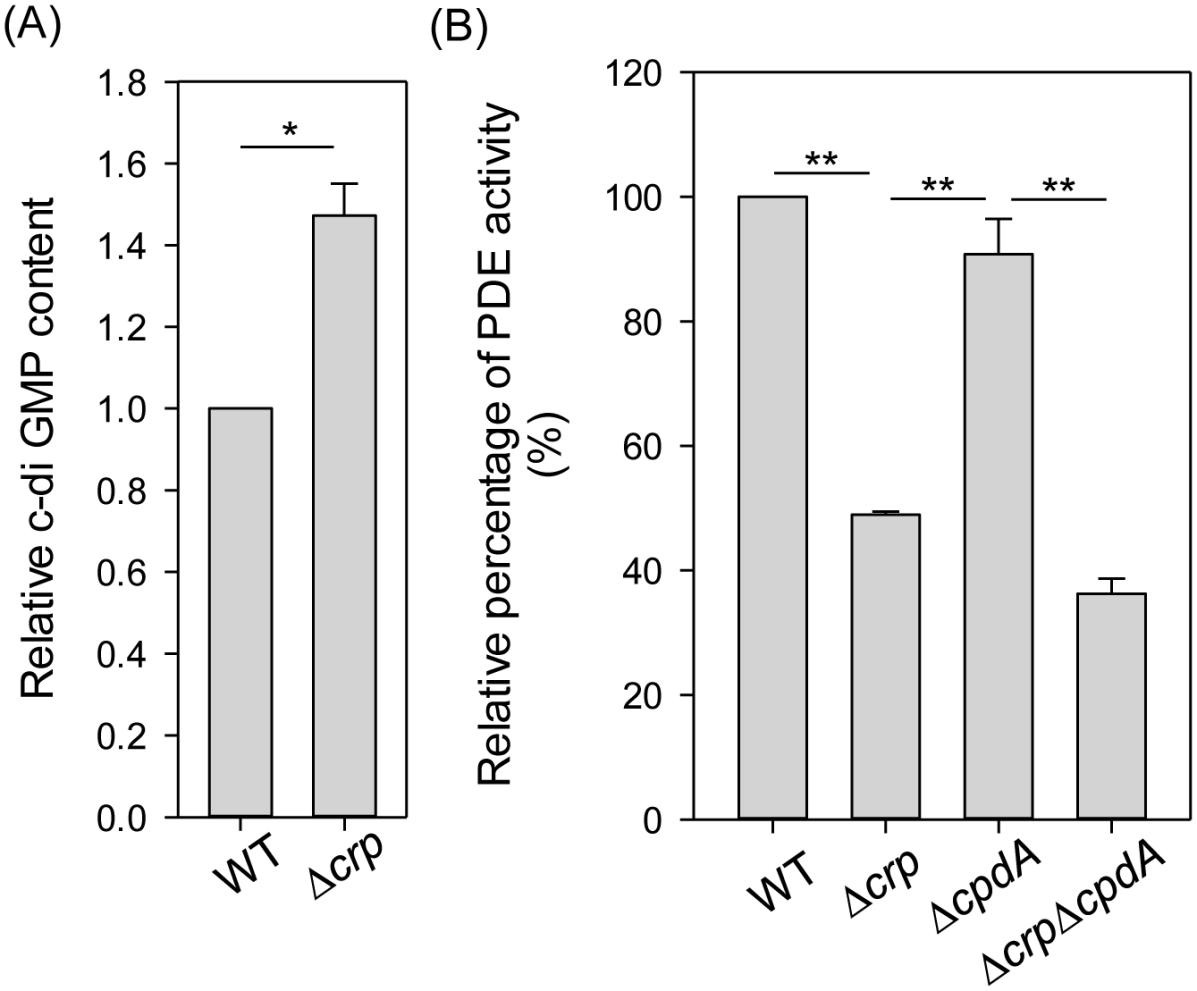
CRP affects the intracellular c-di-GMP amount and the PDE activity. (A) Relative c-di-GMP content of WT and Δ*crp* strains in LB medium was quantified by ELISA according to the manual (Wuhan EIAab Science). (B) The PDE activity of crude extracts of WT and Δ*crp* strains was determined by using bis-*p*NPP as substrate and measuring the absorbance at OD410. Relative percentage of PDE activity was calculated by the OD410 of crude extracts is relative to the OD410 of WT strain. The results are representative of three independent experiments. Error bars indicate standard deviations.

To further investigate the possibility that CRP affects the production of c-di-GMP to inhibit the autoregulatory activity of MrkH and MrkI, the intracellular concentration of c-di-GMP was measured in the WT and Δ*crp* strains. As shown in Fig 6A, the deletion of *crp* in *K*. *pneumoniae* resulted in a higher intracellular c-di-GMP concentration relative to that in the WT strain, confirming that CRP represses c-di-GMP production. In bacteria, the intracellular concentration of c-di-GMP is controlled by DGCs and PDEs [35, 36]. To verify this, the *in vitro* PDE activity in crude extracts of the WT and Δ*crp* strains was first evaluated, using the synthetic substrate bis-*p*NPP in a colorimetric assay. As shown in Fig 6B, the PDE activity in the Δ*crp* extract was significantly lower than that in the WT extract. To rule out the cAMP PDE effect, the PDE activity was further evaluated in Δ*cpdA* and Δ*crp*Δ*cpdA* strains. The absence of *crp* in Δ*crp*Δ*cpdA* strain caused a decrease in PDE activity relative to that in the Δ*cpdA* strain. This indicates that the deletion of *crp* decreases the PDE activity, thereby elevating the intracellular concentration of c-di-GMP and resulting in high expression levels of MrkH and MrkI and hence type 3 fimbriae.

### Effect of CRP on the expression of c-di-GMP-related genes

By analysis of the published genomic sequence of *K*. *pneumoniae* CG43 in the Ensembl Bacteria (http://bacteria.ensembl.org/index.html) database, 11 open reading frames (ORFs) containing the GGDEF domain, 10 ORFs containing the EAL domain, one ORF containing the HD-GYP domain, and six ORFs encoding proteins with both domains (GGDEF and EAL) were found (S1 Fig). In addition, three c-di-GMP receptor proteins harboring a PilZ domain were found in strain CG43; namely, MrkH and the cellulose synthases BcsA-1 and BcsA-2 (S1 Fig). Recently, a new c-di-GMP binding domain, GIL, was identified in enterobacteria [50]. In *K*. *pneumoniae* CG43, BcsE carrying GIL domain was also identified (S1 Fig). To further investigate the role of CRP in the c-di-GMP signaling pathway, the putative CRP binding site was used to search the promoter sequence of these c-di-GMP-related genes, with the maximum number of possible mismatched nucleotides set at 2. Using this criterion, the typical CRP binding site was found upstream of four ORFs containing the GGDEF domain, four ORFs containing the EAL domain, and one ORF containing the GIL domain (Table 1). It suggested that CRP affects the expression of these c-di-GMP-related genes to control the intracellular concentration of c-di-GMP in *K*. *pneumoniae* CG43. To confirm this possibility, the mRNA expression levels of these c-di-GMP-related genes were quantified in the WT and Δ*crp* strains. As shown in Table 1, the expression levels of the genes (locus tag number: D364_04720, D364_08130, D364_13295, D364_22720, and D364_19875) appeared to be increased by more than two-fold in the Δ*crp* strain, indicating that CRP may affect the expression of c-di-GMP-related genes to further influence the c-di-GMP signaling pathway.

**Table 1:** Analysis of CRP binding site in the upstream sequences of the c-di-GMP related ORFs in *K*. *pneumoniae* CG43 genome.

Domain	Locus tag	Gene name	Typical CRP binding site TG(T/C)GA-N6-TC(A/G)CA	Position^a^	Ratio^b^ Δ*crp*/WT
GGDEF domain					
	D364_04720	-	TCAGA-TTGAAC-TCACA	-290 to -275	2.71±0.42
	D364_06045	-	TGCAA-GGAAAT-TCACG	-88 to -73	1.06±0.15
	D364_09195	-	TGCGC-GCAAAG-TCACC	-175 to -160	0.72±0.11
	D364_15015		GGTGA-GAATTG-TCCCA	-96 to -81	1.78±0.82
EAL domain					
	D364_06025	-	TTTGA-TTTTTA-TTACA	-290 to -275	1.83±0.31
	D364_08130	-	AGTGG-AGGGAA-TCACA	-162 to -147	4.61±0.71
	D364_13295	-	TGCCA-CCTGGT-TCTCA	-112 to -97	2.45±0.23
	D364_22720	*yjcC*	TGTGA-ACTATA-TCACA	-382 to -367	2.85±0.24
GIL domain					
	D364_19875	*bcsE*	TGTCA-ATCAGG-GCGCA	-81 to -66	3.84±0.16

^a^Distance to the translational start codon

^b^Mean expression ratio of *crp* mutant relative to wild-type parental strain CG43S3

## Discussion

Diabetic patients have been reported to have a higher susceptibility to infections [51, 52]. *K*. *pneumoniae* strains are more virulent in diabetic than in normal mice has been demonstrated [53]. We had previously found that exogenous glucose could stimulate CPS production in *K*. *pneumoniae* via regulation by the CRP-cAMP signaling pathway [13]. Likewise, this study has demonstrated that could increase type 3 fimbriae expression via the same signaling pathway (Figs 1 and 2). The negative regulation of CRP-cAMP on type 3 fimbriae expression correlated with reductions in the intracellular c-di-GMP concentration and MrkH and MrkI autoregulatory activity (Figs 3 to 5). These findings imply that in response to elevated blood glucose levels in diabetic patients, *K*. *pneumoniae* could increase the expression of virulence factors via the coordination of different regulatory mechanisms for a successful infection.

Several studies have reported that external glucose and the CRP-cAMP signaling pathway could influence fimbriae expression in bacteria [26, 54–56]. In *E*. *coli*, glucose could induce type 1 fimbriae expression and repress P fimbriae expression [26, 55]. Likewise, glucose induced type 1 fimbriae expression in *Salmonella typhimurium* and *S*. *marcescens* [54, 56]. Moreover, the deletion of *crp* in the uropathogenic *E*. *coli* isolate J96 caused a higher expression of type 1 fimbriae, as assessed by mannose-sensitive yeast agglutination (MSYA) assay [26]. However, in *K*. *pneumoniae* CG43, deletion of *crp* did not alter the bacterial MSYA activity (data not shown), suggesting that CRP does not regulate the expression of type 1 fimbriae in this bacterium, at least under this assay condition. Besides this, crosstalk regulation between type 1 and 3 fimbriae expression has been demonstrated in *K*. *pneumoniae* CG43 [29], and whether or not glucose is involved in this regulatory circuit awaits further investigations. On the other hand, deletion of *crp* from *K*. *pneumoniae* resulted in a reduced growth rate (S2A Fig). Colonies of the Δ*crp* strain appeared relatively translucent and small as compared to that of the WT strain (S2B Fig), which was probably due to the altered production of CPS, but not type 3 fimbriae. We considered that the expression of type 3 fimbriae is not a central factor that determines the morphology of *K*. *pneumoniae* colonies, since no obvious difference in colonial morphology could be found between the WT and Δ*mrkA* strain (data now shown).

Previous studies have demonstrated that, in different bacteria, the CRP-cAMP signaling pathway regulates the expression of fimbriae in different ways [26, 54, 56–61]. In *E*. *coli* J96, CRP-cAMP regulates the expression of type 1 fimbriae by an indirect mechanism that requires the activity of DNA gyrase and leucine-responsive protein (Lrp) [26]. In *E*. *coli* CFT073, CRP-cAMP must bind to the promoter upstream of Lrp site 4 to fully express P fimbriae [61]. In *S*. *marcescens*, CRP-cAMP acts as an indirect repressor of type 1 fimbriae expression [56]. These findings indicate that coordination between the CRP-cAMP signaling pathway and other regulatory mechanisms in the control of fimbriae expression is common. Likewise, we found that CRP-cAMP indirectly represses the expression of type 3 fimbriae in *K*. *pneumoniae* CG43. Deletion of *crp* in *K*. *pneumoniae* CG43 increased the expression of *mrkHI*, thereby activating the expression of type 3 fimbriae (Figs 3 and 4). In addition, previous studies have reported that the expression of *mrkHI* and type 3 fimbriae could be activated by intracellular c-di-GMP and the phosphorylation of MrkI [24, 31]. We also found that the deletion of *crp* increased the intracellular c-di-GMP level (Fig 6A). In addition, the absence of *mrkJ* in Δ*lacZ*Δ*crp*Δ*mrkJ* strain resulted in a higher activity of P_*mrkHI*_ relative to that in Δ*lacZ*Δ*crp* strain (Fig 5), confirming that the intracellular c-di-GMP level is critical for the mediation of *mrkHI* and type 3 fimbriae expression by the CRP-cAMP signaling pathway. Furthermore, whether the phosphorylated state of MrkI is also affected by the CRP-cAMP signaling pathway remains to be verified. On the other hand, the ferric uptake regulator (Fur) has been shown to bind directly to P_*mrkHI*_ and P_*mrkA*_ to mediate type 3 fimbriae expression [24]. However, we suggest that Fur is not involved in the CRP-cAMP regulation of type 3 fimbriae expression, since the expression of *fur* was not influenced by the CRP-cAMP signaling pathway in *K*. *pneumoniae* [47].

The interaction between the CRP-cAMP and c-di-GMP signaling pathways in the control of virulence gene expression has been demonstrated in several bacteria [27, 44]. In this study, we also found interplay of the CRP-cAMP and c-di-GMP signaling pathways in the control of type 3 fimbriae expression in *K*. *pneumoniae* CG43. Furthermore, the addition of glucose (0.5%) to LB broth decreased the intracellular concentration of cAMP (S3A Fig) and the promoter activity of P*crp* (S3B Fig) in *K*. *pneumoniae*; moreover, similar results have been reported in *E*. *coli* [62]. On the other hand, the intracellular concentration of c-di-GMP was found to be increased in response to glucose-rich environment (S3C Fig). Therefore, glucose availability is a critical environmental signal to affect CRP-cAMP and c-di-GMP signaling pathways in *K*. *pneumoniae*. In addition, the intracellular c-di-GMP level and PDE activity in *K*. *pneumoniae* were affected by the deletion of *crp* (Fig 6). In the genome of *K*. *pneumoniae* CG43, the search for typical CRP binding sites in the upstream region of ORFs encoding EAL domain proteins revealed three ORFs (D364_08130, D364_13295, and D364_22720) that may be directly repressed by CRP. Depending on various cellular conditions, dual activities or only single activity is present in hybrid proteins with both GGDEF and EAL domains [37, 63]. In *K*. *pneumoniae*, CRP may indirectly affect the expression of ORFs encoding GGDEF proteins or hybrid proteins with both GGDEF and EAL domains, to modulate the intracellular c-di-GMP level and PDE activity. In addition, we found that the mRNA level of D364_04720, encoding a GGDEF domain protein, was increased in the Δ*crp* strain (Table 1). However, whether CRP regulates the expression of D364_04720 to decrease the intracellular c-di-GMP level remains to be investigated.

In *K*. *pneumoniae*, CPS and adherence factors, including type 1 and type 3 fimbriae, have been demonstrated to play important roles in biofilm formation and pathogenesis, and the expression of these virulence genes is under coordinated regulation [24, 64]. Among the adherence factors, type 3 fimbriae have been described as the major determinant of biofilm formation. Furthermore, type 3 fimbriae also mediate the bacterial adherence to epithelial cells and extracellular matrix proteins [23, 65–67]. The ability of *K*. *pneumoniae* colonization and subsequent persistence in mice was reduced when the type 3 fimbriae expression was abolished [68]. In addition, immunization of mice with purified type 3 fimbriae confers protection against following challenge with virulent *K*. *pneumoniae* [69, 70]. These findings indicate the significance of type 3 fimbriae to the virulence of *K*. *pneumoniae*. In this study, we found that *K*. *pneumoniae* grown in LB broth supplemented with 0.5% glucose (Fig 1A) or with *crp* deleted (Fig 1B) resulted in an increased expression of type 3 fimbriae; however, the biofilm-forming activity was reduced (S4 Fig) and the CPS amount was increased [47]. A previous study also indicated that glucose could repress *K*. *pneumoniae* biofilm formation [71]. Since *K*. *pneumoniae* CPS has been shown to impede the assembly of fimbriae and the activity of biofilm formation [21, 72], we postulate that an increased CPS amount may hinder the adherence activity of type 3 fimbriae, resulting in decreased *K*. *pneumoniae* biofilm formation. It is known that biofilm formation is a complex process with several developmental stages in which bacteria express different genes. We suggested that type 3 fimbriae and CPS may act in different developmental stages to influence *K*. *pneumoniae* biofilm formation. However, how the CRP-cAMP signaling pathway orchestrate the expression of CPS and type 3 fimbriae, in response to exogenous glucose levels, to modulate the bacterial biofilm forming activity remains to be investigated. In addition, we noted that CRP represses the expression of *bcsE*, which encodes a GIL domain protein (Table 1). In enterobacteria, BcsE is required for maximal cellulose production and can bind to c-di-GMP via the GIL domain [50]. As cellulose is one of the extracellular polysaccharides involved in biofilm formation, it is possible that CRP modulates cellulose synthesis to influence biofilm formation through the regulation of *bcsE* expression.

**Fig 7:**
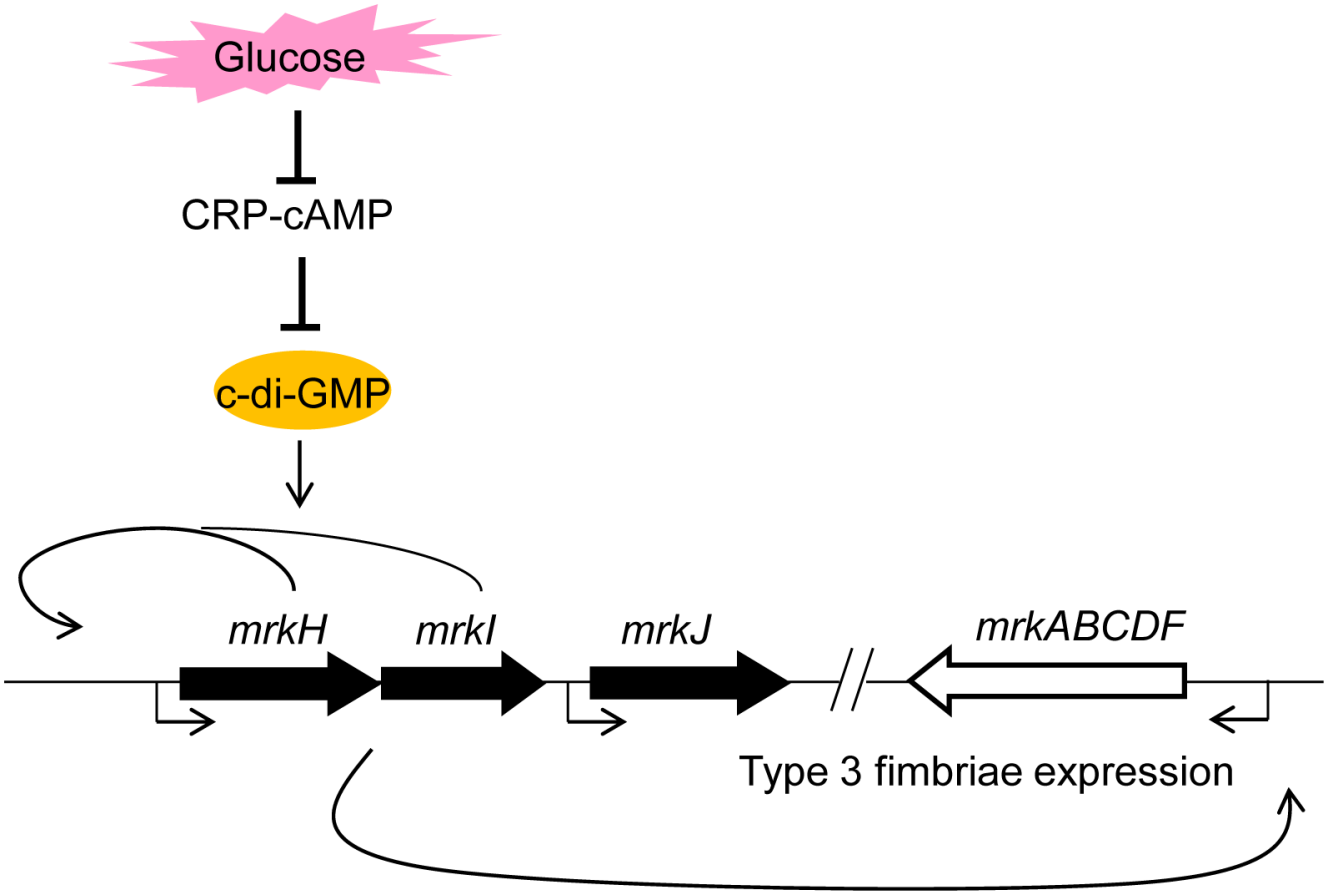
A proposed model for CRP-cAMP signaling pathway on type 3 fimbriae and MrkHI expressions in *K*. *pneumoniae*.

In this study, we have provided important evidence that the CRP-cAMP signaling pathway plays a profound regulatory role in type 3 fimbriae expression in response to an external glucose stimulus, and the regulatory mechanism is also required for MrkH and MrkI activity. In addition, we found that CRP increased the intracellular PDE activity in *K*. *pneumoniae* to further affect the intracellular c-di-GMP concentration. We also found that CRP affects the expression of several c-di-GMP-related genes to influence the c-di-GMP signaling pathway. According the results in this study, we proposed a working model as shown in Fig 7. In *K*. *pneumoniae*, external glucose stimulus repressed the function of CRP-cAMP to further elevate the intracellular concentration of c-di-GMP and the expression of MrkH and MrkI, resulting in a high expression of type 3 fimbriae. To the best of our knowledge, this is the first study to show the coordination between CRP-cAMP and c-di-GMP signaling in modulating type 3 fimbriae expression. Furthermore, we have previously demonstrated that CPS biosynthesis of *K*. *pneumoniae* was affected by CRP-cAMP signaling pathway [47] and a c-di-GMP related protein, YjcC [73]. The expression of *yjcC* may be directly repressed by CRP (Table 1). Therefore, we considered that *K*. *pneumoniae* modulates its virulence gene expression via the interplay of the CRP-cAMP and c-di-GMP signaling pathways in response to exogenous glucose, which may have a major impact on the bacterial virulence in diabetes mellitus patients.

## Supporting Information

S1 Fig Domain architecture of putative c-di-GMP signalling protein encoded by the *K*. *pneumoniae* CG43 genome. The locus tag (D364_number) of the genes encoding (A) GGDEF, (B) EAL, (C) HD-GYP, (D) GGDEF and EAL, (E) PilZ, and (F) GIL domain proteins were indicated.
(TIF)

S2 Fig Deletion effect of *crp* on growth rate and colony morphology. (A) Growth rate of WT and Δ*crp* strain was determined in LB broth for 12 h at 37°C. (B) Colony morphology of WT and Δ*crp* strain was observed on LB plate after 16 h at 37°C.
(TIF)

S3 Fig Effect of glucose on cAMP level, the promoter activity of *crp*, and c-di-GMP level in *K*. *pneumoniae*. (A) Relative cAMP content of *K*. *pneumoniae* CG43S3 in LB medium without or with 0.5% glucose was quantified by cAMP XP^™^ Assay Kit according to the manual (Cell Signaling Technology, Inc). (B) β-galactosidase activities of *K*. *pneumoniae* CG43S3Δ*lacZ* carrying the reporter plasmid p*crp*Z15 (P*crp*::*lacZ*) was determined using log-phase cultures grown in LB medium without or with 0.5% glucose. (C) Relative c-di-GMP content of *K*. *pneumoniae* CG43S3 in LB medium without or with 0.5% glucose was quantified by ELISA according to the manual (Wuhan EIAab Science). The results are representative of three independent experiments. Error bars indicate standard deviations. ** *P* < 0.01 compared to the indicated group.
(TIF)

S4 Fig Effect of glucose and deletion of *crp* on biofilm formation. Biofilm formation of *K*. *pneumoniae* CG43S3 WT or Δ*crp* strain was determined in LB broth supplemented with the indicated glucose concentration using crystal violet staining as previously described [24]. The results are representative of three independent experiments. Error bars indicate standard deviations. ** *P* < 0.01 compared to the indicated group.
(TIF)

S1 Table Bacterial strains and plasmids used in this study. (DOCX)

S2 Table Primers used in this study. (DOCX)
