# Supplementary figures and images for: CRP-Cyclic AMP Regulates the Expression of Type 3 Fimbriae via Cyclic di-GMP in Klebsiella pneumoniae
Source: PLoS One. 2016 Sep 15;11(9):e0162884. doi: 10.1371/journal.pone.0162884 (PMC5025149; doi:10.1371/journal.pone.0162884)

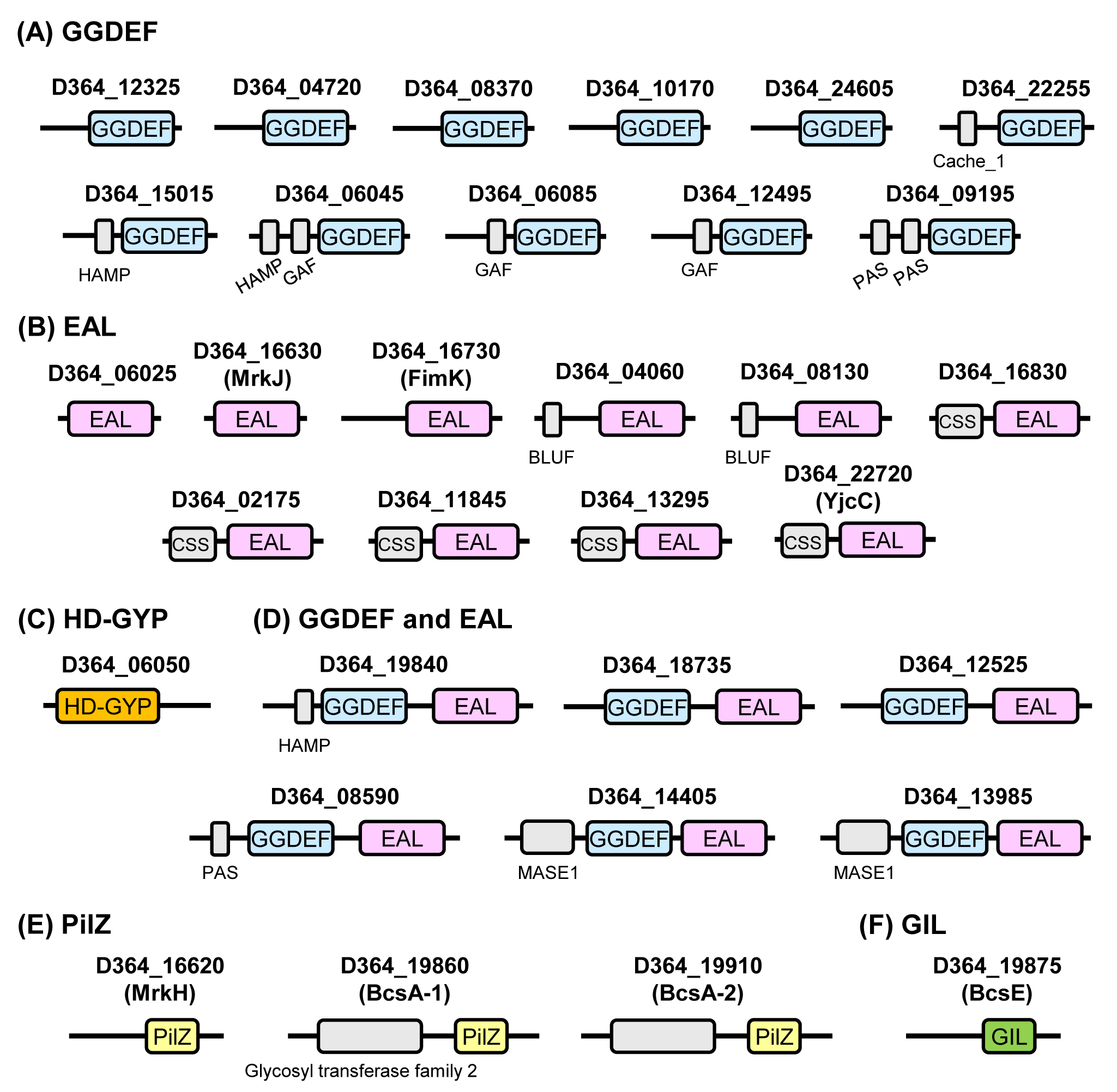

Supplement: S1 Fig — The locus tag (D364_number) of the genes encoding (A) GGDEF, (B) EAL, (C) HD-GYP, (D) GGDEF and EAL, (E) PilZ, and (F) GIL domain proteins were indicated. (TIF) [file pone.0162884.s001.tif]

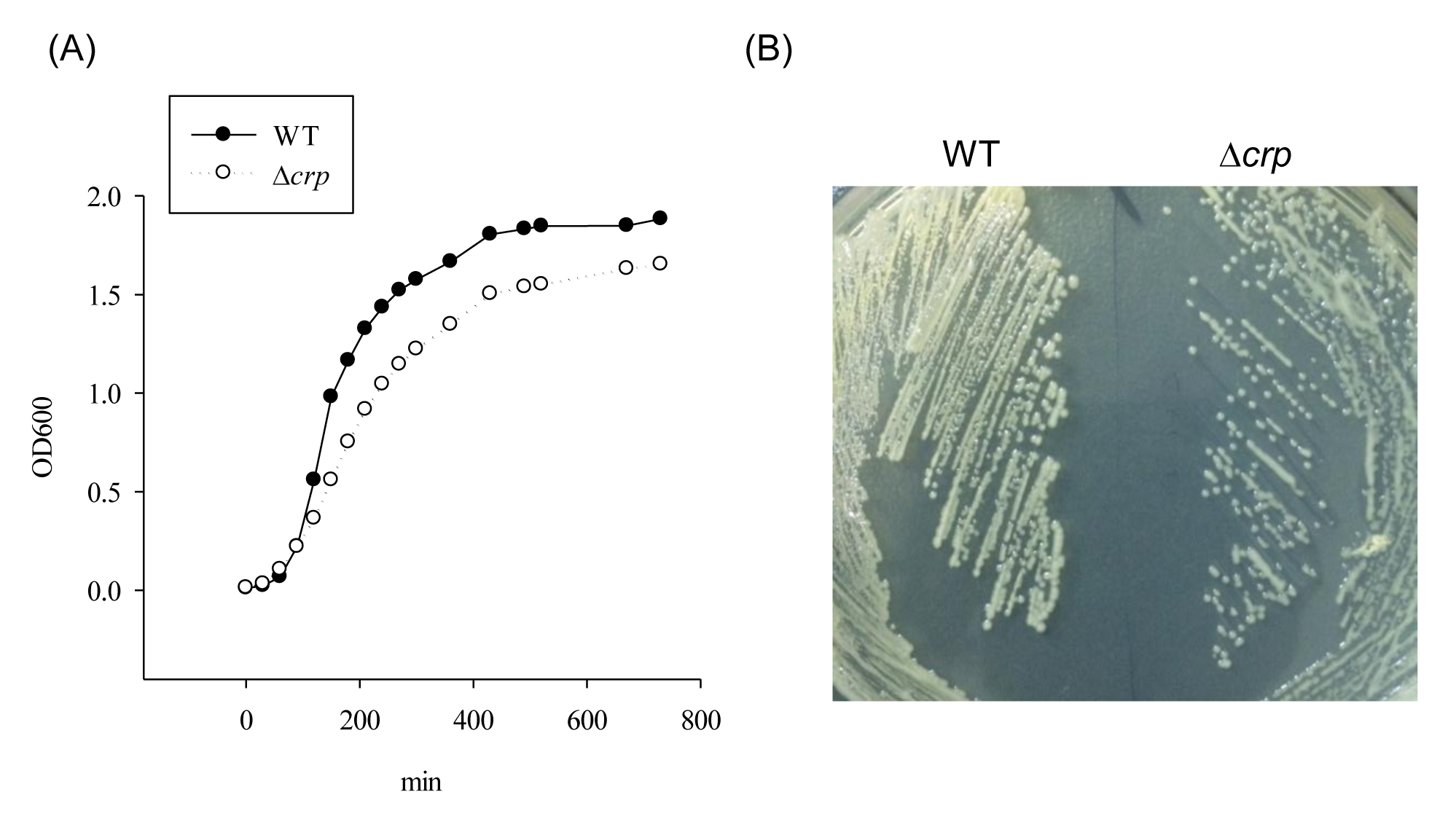

Supplement: S2 Fig — (A) Growth rate of WT and Δcrp strain was determined in LB broth for 12 h at 37°C. (B) Colony morphology of WT and Δcrp strain was observed on LB plate after 16 h at 37°C. (TIF) [file pone.0162884.s002.tif]

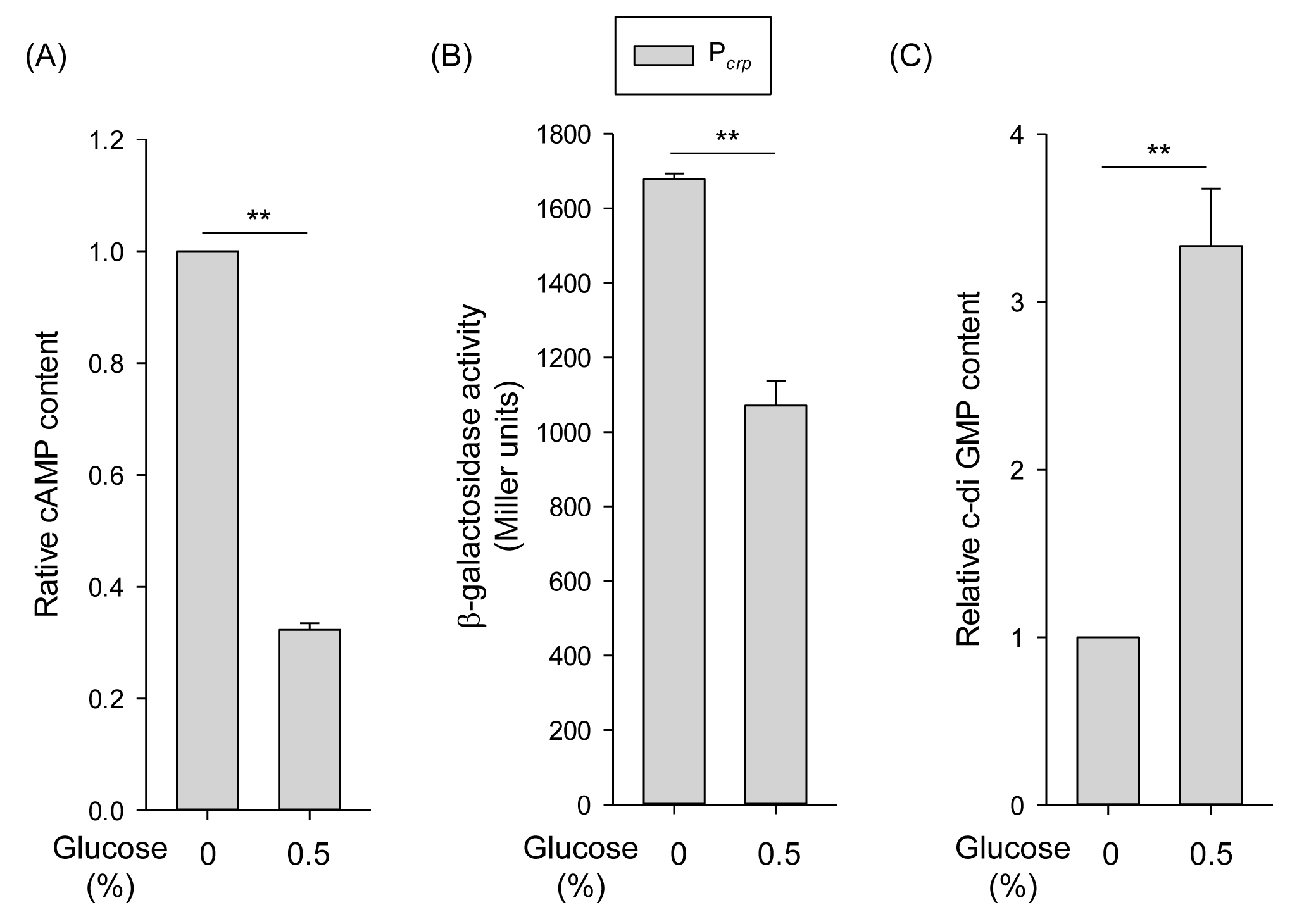

Supplement: S3 Fig — (A) Relative cAMP content of K. pneumoniae CG43S3 in LB medium without or with 0.5% glucose was quantified by cAMP XP™ Assay Kit according to the manual (Cell Signaling Technology, Inc). (B) β-galactosidase activities of K. pneumoniae CG43S3ΔlacZ carrying the reporter plasmid pcrpZ15 (Pcrp::lacZ) was determined using log-phase cultures grown in LB medium without or with 0.5% glucose. (C) Relative c-di-GMP content of K. pneumoniae CG43S3 in LB medium without or with 0.5% glucose was quantified by ELISA according to the manual (Wuhan EIAab Science). The results are representative of three independent experiments. Error bars indicate standard deviations. ** P < 0.01 compared to the indicated group. (TIF) [file pone.0162884.s003.tif]

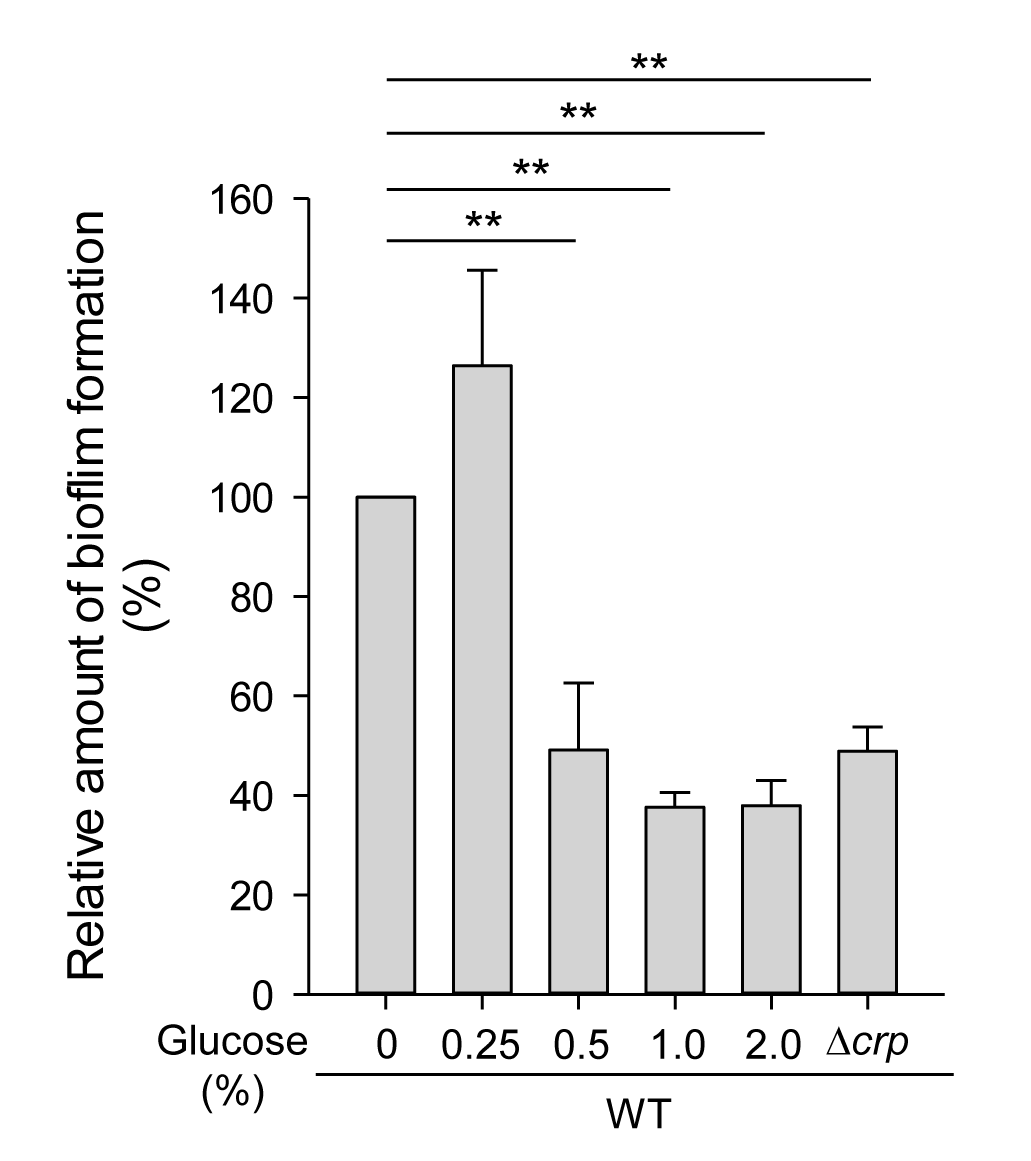

Supplement: S4 Fig — Biofilm formation of K. pneumoniae CG43S3 WT or Δcrp strain was determined in LB broth supplemented with the indicated glucose concentration using crystal violet staining as previously described [24]. The results are representative of three independent experiments. Error bars indicate standard deviations. ** P < 0.01 compared to the indicated group. (TIF) [file pone.0162884.s004.tif]
