# Supplementary material for: CRP-Cyclic AMP Regulates the Expression of Type 3 Fimbriae via Cyclic di-GMP in Klebsiella pneumoniae
Source: PLoS One. 2016 Sep 15;11(9):e0162884. doi: 10.1371/journal.pone.0162884 (PMC5025149; doi:10.1371/journal.pone.0162884)
Supplement: S1 Table — (DOCX) [file pone.0162884.s005.docx]

**S1 Table. Bacterial strains and plasmids used in this study.**

| Strains or plasmids | Descriptions | Reference or source |
| --- | --- | --- |
| *K. pneumoniae* |  |  |
| CG43S3 | CG43 Sm^r^, K2 serotype | [74] |
| ∆*cyaA* | CG43S3∆*cyaA* | [47] |
| ∆*cpdA* | CG43S3∆*cpdA* | [47] |
| ∆*crp* | CG43S3∆*crp* | [47] |
| ∆*crp*∆*cpdA* | CG43S3∆*crp*∆*cpdA* | This study |
| ∆*crp*∆*mrkH* | CG43S3∆*crp*∆*mrkH* | This study |
| ∆*crp*∆*mrkI* | CG43S3∆*crp*∆*mrkI* | This study |
| ∆*crp*∆*mrkJ* | CG43S3∆*crp*∆*mrkJ* | This study |
| ∆*lacZ* | CG43S3∆*lacZ* | [48] |
| ∆*lacZ*∆*crp* | CG43S3∆*lacZ*∆*crp* | [47] |
| ∆*lacZ* ∆*crp*∆*mrkHI* | CG43S3∆*crp*∆*mrkHI* | This study |
| ∆*lacZ* ∆*crp*∆*mrkJ* | CG43S3∆*crp*∆*mrkJ* | This study |
| *E. coli* |  |  |
| DH5α | *supE44*∆*lacU169 (f80 lacZ*∆M15) hsdR *recA1 endA1 gyrA96 thi-1 relA1* | [75] |
| S17-1 *λ pir* | *hsdR recA* *pro* RP4-2 [Tc::Mu; Km::Tn*7*] [*λpir*] | [76] |
| Plasmids |  |  |
| yT&A | Ap^r^, TA cloning vector | Yeastern |
| pACYC184 | Tc^r^Cm^r^, low copy number cloning vector | New England Biolabs |
| pcrp | Cm^r^, 987-bp fragment containing the upstream and coding region of *crp* cloned into pACYC184 | [47] |
| placZ15 | Cm^r^, promoter selection vector, *lacZ*^+^ | [48] |
| pmrkAZ15 | Cm^r^, 402-bp fragment containing the region upstream of *mrkA* cloned into placZ15 | This study |
| pmrkHIZ15 | Cm^r^, 405-bp fragment containing the region upstream of *mrkHI* cloned into placZ15 | This study |
| PmrkJZ15 | Cm^r^, 233-bp fragment containing the region upstream of *mrkJ* cloned into placZ15 | This study |
