# Supplementary material for: CRP-Cyclic AMP Regulates the Expression of Type 3 Fimbriae via Cyclic di-GMP in Klebsiella pneumoniae
Source: PLoS One. 2016 Sep 15;11(9):e0162884. doi: 10.1371/journal.pone.0162884 (PMC5025149; doi:10.1371/journal.pone.0162884)
Supplement: S2 Table — (DOCX) [file pone.0162884.s006.docx]

**S2 Table. Primers used in this study**

| Primer | Sequence (5’🡪3’) | Enzyme cleaved | |
| --- | --- | --- | --- |
| GT284 | GGGATccgctctccatcaatgctaa | *Bam*HI | |
| GT285 | gagaTCTGAAAGACGTAGCGTACAGC | *Bgl*II | |
| GT288 | cggatccAGACAAAATGGAGGGAACCCTA | *Bam*HI | |
| GT289 | cagatcTTACTGGTCTTTATCGTTCCCTC | *Bgl*II | |
| For qRT-PCR | Sequence (5’🡪3’) | TaqMan probes | Target |
| RT11 | ggtaggggagcgttctgtaa | 67 | 23S rRNA |
| RT12 | tcagcattcgcacttctgat |  |  |
| RT29 | taagcaaactgggcgtgaa | 20 | *mrkA* |
| RT30 | tagccctgttgtttgctggt |  |  |
| RT194 | tgaacagcaaaaccgtgatg | 20 | *mrkB* |
| RT195 | gcgccatagtcattaatgttgtta |  |  |
| RT196 | ggtattaccgcgctcaatct | 42 | *mrkC* |
| RT197 | caccgaagaggtatggctgt |  |  |
| RT198 | tatcaaaaccgcggcaac | 70 | *mrkD* |
| RT199 | tcccagtcgtaggaggtgtact |  |  |
| RT200 | gtaacgaaaacgccgggta | 58 | *mrkF* |
| RT201 | cgttagtcgatagggccagata |  |  |
| GT46 | gtttaagttccgccatctcg | 120 | *mrkH* |
| GT47 | ttgcgcttggcttctaagat |  |  |
| GT42 | agttatgccgatgtcatccat | 59 | *mrkI* |
| GT43 | gattctgatggcagaaatatcctt |  |  |
| GT54 | tttcgaggtaaccgaaaacg | 84 | *mrkJ* |
| GT55 | gaggtatcctgtgggctctg |  |  |
| RT177 | atgcgctgtccatgagtatg | 20 | D364_04720 |
| RT178 | ggaagaaaatcattaacgcaaaa |  |  |
| RT181 | agccgctgctctatgctct | 84 | D364_06045 |
| RT182 | ggagacgcttacgcaatatca |  |  |
| RT179 | aagccggttaacggatcg | 70 | D364_09195 |
| RT180 | ccgaagaagtgcatctgaaaa |  |  |
| RT183 | agcgtgataagcgtggtgat | 66 | D364_15015 |
| RT184 | agcgtgaagattgaggctgt |  |  |
| RT159 | ctggctggatgattttggtc | 67 | D364_06025 |
| RT160 | ccactttgacgcaatcgaa |  |  |
| RT161 | cgtaggctggctgatgga | 40 | D364_08130 |
| RT162 | tctgtctcggtgacctcgat |  |  |
| RT163 | tacgggagccatgaatacg | 30 | D364_13295 |
| RT164 | gggtttgctgatatcgatgg |  |  |
| RT175 | aatggtcatccgggaagc | 20 | D364_22720 |
| RT176 | ccgctgaacacaactcacc |  |  |
| RT187 | tccgcctatatgcgtattcc | 70 | D364_19875 |
| RT188 | cgatatgcgcggataaactc |  |  |
